# Is It All About the Form? Norm- vs Criterion-Referenced Ratings and Faculty Inter-Rater Reliability

**DOI:** 10.31486/toj.23.0014

**Published:** 2023

**Authors:** Shannon A. Scielzo, Kareem Abdelfattah, Hilary F. Ryder

**Affiliations:** ^1^ Department of Internal Medicine, University of Texas Southwestern Medical Center, Dallas, TX; ^2^ Department of General Surgery, University of Texas Southwestern Medical Center, Dallas, TX; ^3^ Burnett School of Medicine, Texas Christian University, Fort Worth, TX; ^4^ Internal Medicine Residency Program, Texas Health Harris Methodist Hospital, Fort Worth, TX

**Keywords:** *Criterion-referenced*, *evaluations*, *inter-rater reliability*, *norm-referenced*, *reliability of results*

## Abstract

**Background:** Little research to date has examined the quality of data obtained from resident performance evaluations. This study sought to address this need and compared inter-rater reliability obtained from norm-referenced and criterion-referenced evaluation scaling approaches for faculty completing resident performance evaluations.

**Methods:** Resident performance evaluation data were examined from 2 institutions (3 programs, 2 internal medicine and 1 surgery; 426 residents in total), with 4 evaluation forms: 2 criterion-referenced (1 with an additional norm-referenced item) and 2 norm-referenced. Faculty inter-rater reliability was calculated with intraclass correlation coefficients (ICCs) (1,10) for each competency area within the form. ICCs were transformed to *z*-scores, and 95% CIs were computed. Reliabilities for each evaluation form and competency, averages within competency, and averages within scaling type were examined.

**Results:** Inter-rater reliability averages were higher for all competencies that used criterion-referenced scaling relative to those that used norm-referenced scaling. Aggregate scores of all independent categories (competencies and the items assessing overall competence) for criterion-referenced scaling demonstrated higher reliability (*z*=1.37, CI 1.26-1.48) than norm-referenced scaling (*z*=0.88, CI 0.77-0.99). Moreover, examination of the distributions of composite scores (average of all competencies and raters for each individual being rated) suggested that the criterion-referenced evaluations better represented the performance continuum.

**Conclusion:** Criterion-referenced evaluation approaches appear to provide superior inter-rater reliability relative to norm-referenced evaluation scaling approaches. Although more research is needed to identify resident evaluation best practices, using criterion-referenced scaling may provide more valid data than norm-referenced scaling.

## INTRODUCTION

Graduate medical education programs are required to collect faculty evaluations of resident trainees on a regular basis, but the mechanism by which residents are evaluated is left to the discretion of individual programs. Thus, programs have employed a wide variety of approaches. However, many programs seem to have been influenced by changes in accreditation reporting requirements. For example, in 2013, the Accreditation Council for Graduate Medical Education (ACGME) launched the Next Accreditation System (NAS) that transitioned the focus of resident performance reporting practices toward assessing specific behavioral criteria linked to an underlying competency continuum (ie, milestones). Biannually, programs must indicate each trainee's performance on the milestones, and these milestone reports oftentimes heavily rely on faculty evaluations of resident performance. Some performance management systems facilitated milestone reporting by creating default evaluation scales (based on the milestone reporting structure, with the ratings of “critical deficiencies,” “ready for unsupervised practice,” and “aspirational”) that likely encouraged programs to use these scales. Furthermore, many individuals may have elected to use the same scaling as the milestones to simplify mapping in general.

Thus, resident evaluations from many programs similarly transformed to mirror the biannual ACGME milestone reporting scales in which residents are mapped to where they fall on the ability continuum (criterion-referenced). Prior to this transformation, most programs focused on identifying stragglers or those excelling and used comparison-type (norm-referenced) scales. Little published evidence compares these 2 types of scaling approaches. Studies evaluating the NAS have focused on evidence of the validity or construct validity of milestones ratings^1-3^ or on the feasibility of reporting,^4^ or they have used data gathered through the NAS to stratify competency-based achievement by level of training.^5^ But these studies have not addressed the reliability of evaluation data gathered by programs to support the NAS. This research effort sought to provide some preliminary inferences in this regard.

### Norm- vs Criterion-Referenced Assessments

In the context of evaluation creation, there are many potential considerations. First, you need to make sure that evaluators will be able to interpret the content in the scales (ie, the content is free of jargon; it is free of text that may be interpreted differently based on sex, race, or other unrelated characteristics; and the items measure important behaviors or skill sets). You need to ensure that the numbers you ask raters to use—the scale you have selected—are appropriate for the content being assessed and the proposed application. Scaling drives the translation from an abstract construct (eg, How well does a resident communicate?) to a tangible number. But that number can be more or less accurate depending on several factors (eg, difficulty to use the scale, bad frames of reference).^6^

Norm-referenced evaluation scales require raters to make assumptions regarding where individuals reside on average relative to other similar individuals without specific regard to the competency continuum.^6^ Thus, resultant ratings indicate whether an individual is better, worse, or about the same as a normative referent (such as the average resident).

Glaser and Klaus proposed that norm-referenced scaling was not sufficient for educators’ needs because the actual proficiency level of trainees needs to be identified.^7^ Certain standards have to be met, and only via aligning trainee performance to these standards can educators ensure that trainees are adequately prepared. Thus, criterion-referenced scales assume an underlying continuum of skill to which resident performance can be mapped (eg, from novice to expert, with a clear point at which trainees are ready for unsupervised practice).

**Figure 1.**
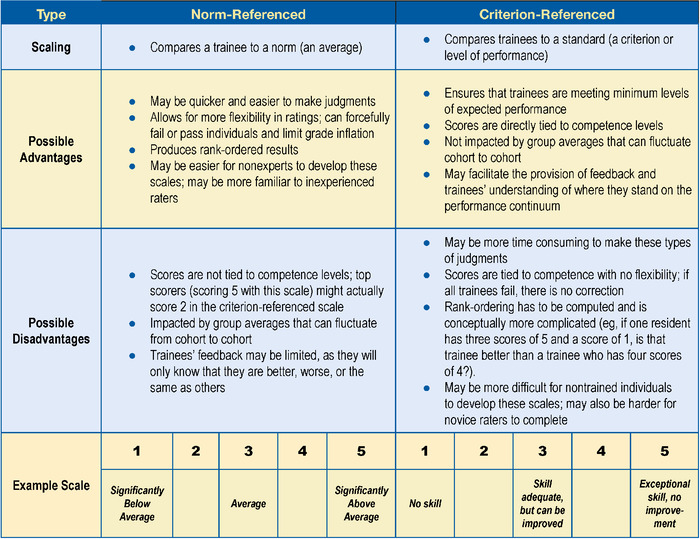
Brief comparison of criterion- and norm-referenced scales.

Arguably, the criterion-referenced scales may remove some ambiguities regarding the comparison group (eg, relative to interns on this rotation, or interns doing the procedures, or all residents on average); however, making norm-referenced comparisons may be less cognitively demanding than making absolute (criterion-based) judgments.^8^ Norm-referenced evaluations may also be easier for nontrained individuals to develop, and they may be more familiar to inexperienced raters.^9^ Figure 1 provides a brief summary of some of the advantages and disadvantages of both types of scaling approaches, and Appendix A provides details on evaluation scaling.

Guidelines to date have mixed inferences at best, with arguments supporting both sides. In the context of education in general, Lok et al suggested that the 2 approaches are to some extent intertwined.^10^ Effective criterion referencing necessitates an understanding of how individuals on average perform to develop the required standards of performance. Thus, norm referencing is inherent in developing the standards in criterion-referenced assessment. Interestingly, Lok et al also argued that norm-referenced scaling can allow for more freedom for evaluators. For example, some trainees can be forced to fail in a norm-referenced model regardless of the overarching quality of performance. Conversely, with a criterion-referenced model, as long as trainees achieve the standards, they pass. Thus, criterion-referenced approaches may increase grade inflation and passing and may not effectively identify the worst performers.^10^ However, others have pointed out that norm-based evaluation approaches may lead to standards being set below where they would with criterion-based approaches.^11^

In the context of medical education, little research has examined the impact of scaling type on assessments. For example, Wittels et al examined the correlation of resident self-assessment and corresponding faculty ratings of clinical competence using milestone ratings in the context of a highly standardized training simulation.^12^ Using 1-way random effects intraclass correlation coefficients (ICCs), they found absolutely no agreement between residents and faculty on these ratings and low agreement between faculty. Even faculty did not highly agree when rating residents using milestones in an ideal testing context of a standardized simulation. Conversely, Scielzo et al found that resident self-reports and faculty reports of resident criterion-assessed competencies were consistent.^13^ This study, however, used a correlational design (thus, statistically assessed consistency instead of agreement). Pereira and colleagues, arguing that undergraduate medical education needs to move away from norm-referenced assessment, suggested that current norm-based approaches are not predictive of later performance and that undergraduate medical education needed to transition to criterion-referenced assessment.^14^ Interestingly, they also noted the dearth of research assessing the impact of criterion-referenced vs norm-referenced assessment in graduate medical education.

Thus, as criterion-referenced evaluations are now widely used in resident education, examining this form of evaluation in comparison to its predecessor is imperative. Given our duty to society to ensure that physician trainees are ready for independent practice upon graduation and to ensure that our inferences in this regard are as accurate as possible, examining reliability is an important first step.

### Inter-Rater Reliability

We elected to examine inter-rater reliability to make some initial inferences regarding the quality of the information gained from the evaluation scaling types. Inter-rater reliability, the consensus or degree of agreement among raters, provides one indicator of the psychometric utility of a scale.^6^ Reliability in evaluation refers to the reproducibility of data or scores over time, event, or items designed to measure the same constructs.^15^ Reliability is an important consideration in evaluating the utility of a given measurement; without reliability, inferences are not stable. In other words, without reliability, you cannot have validity.^16,17^ Imagine your home scale giving the following 3 readings in a 3-minute period: 87 lbs, 205 lbs, and 147 lbs. Because the scale cannot systematically infer a weight, the weight results clearly are not valid, and it is probably time to discard the scale. Appendix B provides general information about reliability.

In the case of resident assessments, low inter-rater reliability might suggest that the evaluation questions were hard to understand, that raters did not actually have enough opportunities to observe rating behaviors, that the items did not provide a good representation of the underlying constructs, or that the scaling approach may not be ideal for the behaviors being measured.

In this study, we sought to determine the effect of using norm-referenced vs criterion-referenced scales on the reliability of evaluation data. Specifically, we examined the impact of evaluation scaling type (ie, criterion-referenced vs norm-referenced) on the inter-rater reliability of faculty members completing resident performance evaluations.

## METHODS

### Setting

We compared the inter-rater reliability of 4 different evaluation forms. These evaluations came from 3 graduate medical education residency training programs at 2 universities: Dartmouth-Hitchcock and the University of Texas Southwestern (UTSW). Two of the evaluations were criterion-referenced and 2 were norm-referenced. One of the criterion-referenced evaluations had an additional norm-referenced item that we also included in our analysis.

Program I was the internal medicine program at Dartmouth-Hitchcock. Program II was the internal medicine program at UTSW. Program III was the general surgery program at UTSW. Program I had both norm-referenced (referred to as norm-referenced 1, n=49) and criterion-referenced (referred to as criterion-referenced 1, n=110) evaluations. The norm-referenced evaluations were collected in 2012-2013 prior to the ACGME transition, and the criterion-referenced evaluations were collected in 2013-2014. Program II used an evaluation form (n=196) that had predominantly criterion-referenced items (referred to as criterion-referenced 2), but also had 1 norm-referenced item (referred to as norm-referenced 2). Program III had a norm-referenced evaluation (referred to norm-referenced 3, n=71). We elected to include all of these different evaluations to try to overcome some of the limitations of this study, such as temporal differences (via collecting asynchronous and synchronous evaluations) and having the exact same raters and individuals being rated (ie, the UTSW criterion-referenced items and also 1 norm-referenced item). Appendix C presents the specific items used in each form and how each was linked to competencies.

### Study Design

This study was an archival data analysis, an examination of end-of-rotation data collected for educational purposes (the required end-of-rotation evaluations). Thus, faculty evaluation behaviors were not influenced by knowing that their scores would be examined. Ethical approval was obtained from the Dartmouth College Institutional Review Board that declared the study exempt from formal review.

Data were obtained for residents in postgraduate years (PGY) 1 to 3 in Programs I and II, and from residents in PGY 1 to 5 in Program III. Evaluation items had been mapped during evaluation creation to their relevant ACGME competencies: Practice-Based Learning and Improvement, Patient Care, Professionalism, Medical Knowledge, Interpersonal and Communication Skills, and Systems-Based Practice. A few items assessed overall perceptions of a resident's standing (Overall).

If multiple items were associated with a competency, an average was computed (see Appendix C for more detail). As an example, if we had 2 items assessing Patient Care, we averaged these 2 items to represent that construct for each evaluation administration (eg, Dr Smith's first evaluation had a 3 for item 1 and a 4 for item 2, resulting in Dr Smith receiving an average of 3.5 for the first evaluation).

The evaluation for Program II was designed with the goal of minimizing the time to complete while retaining as much important information regarding performance as possible. After careful analysis from the development team, we decided that broader items tapping multiple competencies were preferred. Thus, this evaluation contained 2 items that each represented 2 constructs (ie, Patient Care and Medical Knowledge were represented by 1 item, and Professionalism and Systems-Based Practice were represented by 1 item). Thus, the same reliability index was used in the analysis for these competencies (ie, the same for Patient Care and Medical Knowledge, and the same for Professionalism and Systems-Based Practice) for Program II.

### Data Analysis

Inter-rater reliability was assessed with a 1-way random effects model ICC.^18^ This assessment enabled us to examine the correspondence of scores across random raters (any of our faculty members may make these ratings). Similarly, we needed to evaluate whether our raters were in agreement, not just consistent. We also wanted to make estimates for a larger sample of raters consistent with our most important decisions (eg, readiness for graduation).

The first 2 observations for each trainee were used, with a resultant ICC (1,1). These values were then applied to the Spearman-Brown prediction formula^19^ based on an average of 10 assessments per resident, the minimum annual number of evaluations per resident, to provide an estimate of the accuracy of the data used for milestone reporting. This formula allows us to make more accurate estimates of reliability based on applied utilization, rather than just relying on the lower reliability inferences from the 2 observations.

We examined the average for all criterion-referenced ICC values within a competency compared to the average for all norm-referenced values. Thus, all ICCs were averaged within competency and scaling type. To examine the average reliability based on scaling type, we averaged all independent reliability values within scaling type after removing duplicate values for Program II. Appendix B provides general information about ICCs.

To facilitate comparison and the interpretation of CIs, these effect sizes were translated to *z-*scores. The 95% CIs were computed around each *z-*score using 1/sqrt(*N*-3) for the standard error. Inter-rater reliability estimates were calculated using SPSS Statistics version 27.0 (IBM Corporation),^20^ and Spearman-Brown estimates and *z*-score confidence intervals were calculated in Microsoft Excel (Microsoft Corporation).

Finally, all items were averaged for each individual being rated to provide an estimate of their overarching standing based on these evaluations. Thus, for resident X, all items were averaged for both evaluators, creating a composite estimate of overall ability. Then, these composite scores were broken into 5 equal intervals and mapped back to the underlying scales used to rate the trainees, enabling us to examine the distribution of scores for each program with multiple competencies (excluding the 1 norm-referenced assessment item in Program II) and providing another indicator to examine the quality of the data obtained. Graphs were created for each scaling type in Microsoft Excel.

## RESULTS

### Competency Averages

First, we examined the extent of the impact of scaling type (norm-referenced vs criterion-referenced) for each competency. Examining the *z*-score for the average reliability for each scaling type by competency, higher reliability averages were observed for criterion-referenced evaluation scales relative to norm-referenced scales for all competencies (Table). For example, the Interpersonal and Communication Skills competency had an average *z*-score of 1.16 (CI 1.05-1.27) for our criterion-referenced evaluations vs an average *z*-score of 0.76 (CI 0.63-0.89) for the norm-referenced evaluations. Neither *z*-score falls within the CI of the other, and thus, with 95% confidence is statistically different. Furthermore, the criterion-referenced item that assessed overall competence was statistically higher than the average of the 2 items using norm-referenced scaling (*z*=1.83 vs 1.31).

**Table.** Inter-Rater Reliability Estimates for All Program Evaluation Forms and Competency Areas

	Program and Form Type													
	Program I: Criterion-Referenced		Program II: Criterion-Referenced		Criterion-Referenced Average		Program I: Norm-Referenced		Program II: Norm-Referenced		Program III: Norm-Referenced		Norm-Referenced Average	
Competency	*Z*-Score	95% CI	*Z*-Score	95% CI	*Z*-Score	95% CI	*Z*-Score	95% CI	*Z*-Score	95% CI	*Z*-Score	95% CI	*Z*-Score	95% CI
Items Assessing Overall Competence			**1.83**	1.69-1.97	**1.83** ^a^	1.69-1.97	**1.47**	1.18-1.76	**1.19**	1.05-1.33			**1.31**	1.20-1.42
Interpersonal and Communication Skills	**0.83**	0.64-1.02	**1.95**	1.80-2.09	**1.16** ^a^	1.05-1.27	**0.55**	0.26-0.84			**1.05**	0.81-1.28	**0.76**	0.63-0.89
Patient Care	**(1.59)**	1.40-1.78	**1.83** ^b^	1.69-1.97	**1.66** ^a^	1.55-1.73	**(1.19)**	0.90-1.48			**0.97**	0.74-1.21	**1.07**	0.89-1.25
Practice-Based Learning and Improvement	**1.42**	1.23-1.61	**1.95**	1.80-2.09	**1.62** ^a^	1.51-1.73	**0.78**	0.49-1.06			**0.81**	0.57-1.05	**0.79**	0.61-0.97
Professionalism	**0.95**	0.76-1.14	**1.66** ^b^	1.52-1.80	**1.20** ^a^	1.09-1.31	**0.37**	0.08-0.65			**0.87**	0.63-1.11	**0.58**	0.40-0.76
Medical Knowledge	**1.47**	1.28-1.66	**1.83** ^b^	1.69-1.97	**1.52** ^a^	1.41-1.63	**1.42**	1.13-1.71			**(1.05)**	0.81-1.28	**1.20**	1.02-1.38
Systems-Based Practice	**(1.07)**	0.88-1.26	**1.66** ^b^	1.52-1.80	**1.29** ^a^	1.18-1.40	**0.54**	0.25-0.82			**1.00**	0.76-1.23	**0.73**	0.55-0.91

^a^The average of form type across programs is statistically higher (ie, all averages of criterion-referenced evaluations were higher than norm-referenced evaluations, as also indicated by nonoverlapping CIs).

^b^One item represented 2 competencies.

Notes: Values in parentheses are based on an average of items. Larger *z*-scores indicate higher inter-rater reliability.

### Scaling Type Averages

We averaged independent individual scores of a type to make inferences about the impact of scaling type, independent of competency, on reliability. For the criterion-referenced average, for Program I, all 6 competencies (Interpersonal and Communication Skills, Patient Care, Practice-Based Learning and Improvement, Professionalism, Medical Knowledge, and Systems-Based Practice) plus the question assessing overall competence were averaged. For Program II, the 4 independent competencies (Interpersonal and Communication Skills, Practice-Based Learning and Improvement, and the Patient Care/Medical Knowledge and Professionalism/Systems-Based Practice combined indices) plus the question assessing overall competence were averaged. The norm-referenced average included all 6 competencies plus the question assessing overall competency for Program I, the item assessing overall competence for Program II, and all 6 competencies for Program III. Overall, criterion-referenced scaling (*z*=1.37, CI 1.26-1.48) had statistically higher reliability than norm-referenced scaling (*z*=0.88, CI 0.77-0.99) (data not included in the Table).

### Competencies Within Scaling Type

All criterion-referenced scale *z*-scores trended toward being higher than their corresponding norm-referenced values within each competency, except for Interpersonal and Communication Skills. For this competency, the criterion-referenced score of 0.83 in Program I was lower than the norm-referenced score of 1.05 in Program III.

Statistically, the Program II scores were more reliable than the Program I scores for all competencies in the criterion-referenced scaling evaluations. In general, however, the individual criterion-referenced evaluations provided higher inter-rater reliabilities than the norm-referenced assessments.

### Distributions of Composite Scores

**Figure 2.**
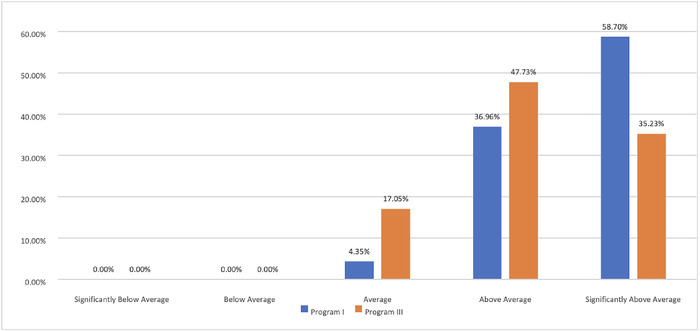
Response distributions for competency and rater composite scores for norm-referenced scales.

**Figure 3.**
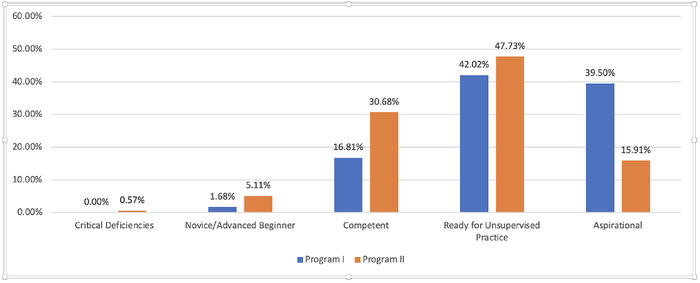
Response distributions for competency and rater composite scores for criterion-referenced scales.

We examined the response distributions for aggregate composites of competencies, based on evaluation scaling type. The majority of scores for the norm-referenced scales were in the above-average range or higher, with no composite scores below average (Figure 2). The criterion-referenced scales also had a majority of items in the second quintile (ready for unsupervised practice) but also had a substantial number in the lower quintiles, even some in the fifth quintile (critical deficiencies) for 1 of the evaluation forms (Figure 3). The distributions appeared relatively similar within scaling type, and the criterion-referenced scaling appeared to produce scores that were more varied (ie, used the lower end of the response continuum).

## DISCUSSION

The 2013 transition of the ACGME changed the way that many graduate medical education programs evaluate their trainees. The milestone-based approach requires faculty educators to use a criterion-referenced approach to evaluate candidates. Little is known about the impact of this transition,^14^ but many programs appear to have changed their evaluations to align with the milestone structure. The results of this study suggest that this transition has likely had a positive outcome on at least the reliability of evaluations. Specifically, criterion-referenced scaling in general results in higher inter-rater reliability than the previously used norm-referenced scaling approaches.

Comparing the average reliability within scaling type within each competency, all 6 competencies and the items assessing overall competence were statistically superior for the criterion-referenced evaluations relative to the norm-referenced evaluations. Furthermore, examining the average of all different evaluations across competencies within scaling type, criterion-referenced scaling was superior.

Not surprisingly, given the variation of evaluations used by the programs, individual evaluations demonstrated some differences. However, criterion-referenced evaluations performed better on average than the norm-referenced evaluations. More research is needed to explain some of these other differences.

As noted by Abdel-Aziz et al,^21^ resident assessment is extremely complex and should incorporate many different indices in addition to faculty evaluations. However, each information source must be as valid as possible to ensure that resident experiences are tailored appropriately to optimize their skill set development. As previously noted, higher reliability for our evaluations enables us to have higher accuracy and is an important first step in the process of evaluating the quality of our assessments.

Furthermore, in the examination of the underlying distributions of our composite scores across competencies, criterion-referenced scales appear to produce more variability. The norm-referenced composites were all at the midpoint or above, whereas the criterion-referenced scaling produced scores at the lower ends of the continuum. It is conceptually impossible for everyone to be average or above, yet our results are consistent with other studies.^22-24^ Thus, the increased variability demonstrated by criterion-referenced scaling also likely reflects a better measurement approach.

### Value of Feedback to Guide Trainee Development

In addition to the quality of the data obtained, the utility of the evaluations for faculty and trainees should also be considered. Faculty feedback is well-established as an extremely important component of trainee development. Faculty feedback helps guide energy, correct mistakes, highlight strengths, and provide necessary resources for trainee success.^25,26^ The evaluations we use should support this endeavor by highlighting the educational components we expect trainees to learn. These evaluations should also delineate expected levels of performance and provide a clear frame of reference for faculty and trainees. Translating scores into meaningful metrics with specific behaviors can help faculty communicate developmental opportunities and can also allow trainees to clearly see where they stand. In other words, criterion-referenced scaling is likely superior for trainees as they will learn what specific standards they are not achieving, rather than receiving an evaluation such as “the other 4 trainees in the program did better than you.” Trainees can achieve acceptable levels of performance for each area and then focus on excelling where they can truly be great, rather than potentially failing because they were a little too far below some superstars in 1 or more areas. Thus, criterion-referenced scaling likely offers many benefits in the provision of feedback and in our goal to optimize the development of robustly competent physicians.

### High-Value Evaluations

As a final point, optimizing the utility of our efforts whenever possible is important. As faculty educators and administrators, we are oftentimes seemingly overtasked and under-resourced. An internal study at UTSW that was conducted in 2018 by the Graduate Medical Education Office found that the majority of faculty reported feeling overwhelmed with evaluations. Faculty also felt that the effort they put into the evaluations was not leading to needed change.

We should be maximizing the return on investment. Evaluating the quality of our assessments is important to ensure we are effectively using our time in addition to ensuring that we are appropriately training and evaluating our trainees. Thus, we need to strive for high-value evaluations. Just as we strive to provide high-value care to our patients, we must also create and oversee high-value evaluations for our educational systems.

### Limitations and Future Research

Several limitations should be noted. First, this is a preliminary study based on archival data. Different study designs, such as using controlled simulation environments, controlling for specific faculty members’ ratings, and integrating other sources of data, are needed. Furthermore, generalizability is limited given that only 3 programs participated. Future studies need to examine a broader range of programs—including smaller fellowship programs—and help identify the factors that are impacting our inferences.

We had very different items across our samples. We attempted to map them to their overlying competencies, but multiple items had to be averaged in some cases, and in other cases we had a single item representing multiple competencies. Future efforts should standardize the items and have discrete items for each content domain. Moreover, data were collected at different points in time. We tried to indirectly address some of these issues with a variety of approaches (eg, concurrently collected types within the same sample, different programs), but more research is needed.

Future research should examine other validity and reliability estimates, such as the correspondence of ratings with in-service/board examination scores and/or other indicators of performance. Future research needs to evaluate faculty satisfaction with these rating types (eg, perceived utility, ease of use). Given the importance of our evaluation efforts, much more research is needed to identify best practices for creating the most psychometrically and practically useful evaluation tools possible.

## CONCLUSION

We found that criterion-referenced scaling results in higher inter-rater reliability relative to norm-referenced scaling for faculty evaluations of resident performance. Although more research is needed, criterion-referenced scaling appears to provide better inter-rater reliability and, in turn, more valid inferences for deriving milestone data. The results of this study suggest that programs should consider adopting criterion-referenced scaling approaches to ensure that programs are making the most accurate inferences possible when assessing residents’ skills.

## Figures and Tables

**Figure A1.**
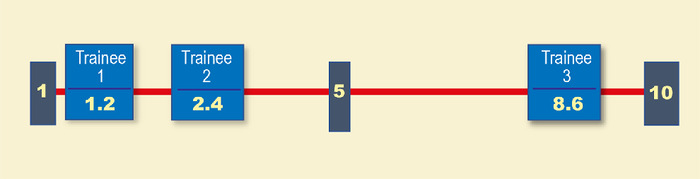
Example trainees scaled on a continuum.

**Figure A2.**
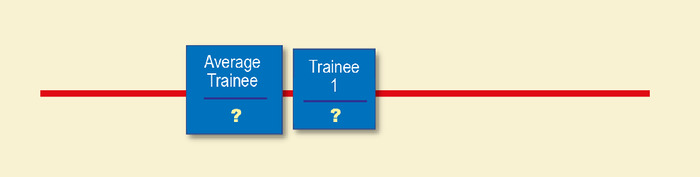
Example norm-based comparison.

**Figure A3.**
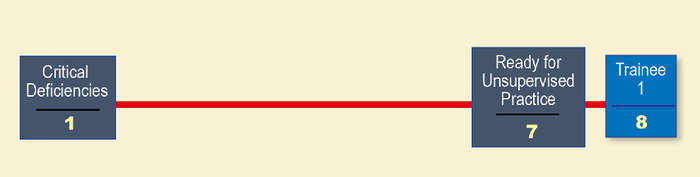
Example criterion-based comparison.

## Notes

*This article meets the Accreditation Council for Graduate Medical Education and the American Board of Medical Specialties Maintenance of Certification competencies for Systems-Based Practice and Practice-Based Learning and Improvement.*

## Appendix A. Evaluation and Scaling

In evaluation development, one of the most important decisions to make is regarding the scaling. In simplest terms, scaling refers to the type of ruler or scale used to measure the content of interest.^1^ For example, how will we apply a ruler to an abstract construct such as happiness? Will we have people push a button every time they feel happy throughout a week, and then score every hour without a button push as 0 and every hour with a button push as 1? Or will we ask people how they currently feel in reference to a 10-point happy face scale? One or the other approach may be better, depending on our goals.

As another example, we might be curious to know which of the vegetables in our kitchen weighs more as we prepare to make a meal. Does the zucchini or the eggplant weigh more? If we have an old-fashioned balance scale with 2 hanging baskets, we can drop a vegetable on each side to see which drops down lower. We could create a contraption using a fulcrum and attempt to place the vegetables equidistant from the center. These approaches can tell us which vegetable is heavier, but we will not know their specific weights. A digital kitchen scale can provide each vegetable's weight, from which we can infer which is heavier. All 3 examples are measurement scales, but we obtain a different level and a potentially different quality of data from each of them.

Different approaches and the quality of the data obtained from different scaling approaches have been evaluated with extensive psychometric research.^1^ When working with measurement of difficult constructs, such as evaluating the skills of resident physicians, considerations oftentimes include the way the data will be used (eg, What decisions will be informed by the data?), the characteristics of the raters (eg, Are raters well-trained experts or just generally familiar with what they are rating? Do they have the cognitive ability to evaluate the content?), and the difficulty of the content to be evaluated (eg, Are we asking raters to check *yes* or *no* to whether a resident introduced themselves when entering a patient room, or are we asking them to do something more complicated like assess an individual's emotional intelligence responding to a series of complicated scenarios in which multiple sources of information are integrated?).^2^ Thus, many considerations go into choosing the best scaling approach for an evaluation instrument.

One approach suggested by Thurstone (1927) is compared to the Weber law.^3^ Thurstone presents what he refers to as a new psychophysical law that he called the law of comparative judgment. This approach necessitates that every possible individual targeted for rating is evaluated, and across these pairwise comparisons we can determine exactly where on a continuum each rated individual falls. Thus, if we have 4 trainees in a program, we would need (n-1)/2 = (4-1)/2 = 6 comparisons (trainee 1 vs 2, trainee 1 vs 3, trainee 1 vs 4, trainee 2 vs 3, trainee 2 vs 4, and trainee 3 vs 4) from an evaluator. For a large program (such as a program with 150 residents), we would need 75 ratings from an evaluator. This approach assumes that the rater is accurate. Moreover, the approach is not concerned with the quality of raters but just with identifying where a rated individual falls on a continuum (Figure A1).

Few individuals would be able to provide this many ratings in a meaningful way. We might elect to make aggregate comparisons (ie, use a norm-based approach). With this scaling method, we ask faculty members to compare a trainee against all others. However, as shown in Figure A2, when a rater compares trainee 1 against the average trainee, the rater does not have a specific number to apply to the trainee or to the average trainee. As many of us have observed, faculty raters oftentimes rate all candidates above average,^4-6^ which raises some concern about the accuracy of these types of ratings.

Given this lack of grounding to a number, the resultant data are commensurate with race rankings to some extent. If we just focus on ranking (first place, second place, third place), we lose extremely important information. For example, maybe the difference between the first and second positions was 1 second, whereas the difference between the second and third place positions was 15 minutes. Thus, this approach is much easier than a comparative judgment approach if we only need ranking data. However, in many cases, ranking data alone without sophisticated corrections or metrics (eg, the timer data) may not be very informative.

Moreover, with this ordinal ranking approach, we do not necessarily know where any given faculty member feels that the referent (eg, the average trainee) falls on the continuum. Faculty member A may feel that the average trainee is at about 4 on the underlying performance continuum, whereas faculty member B may believe that the average trainee falls at a 7 on the same continuum. Thus, knowing how someone stands against an average gives us insight into their actual level of skill.^2,7^ Further confounding matters, faculty member A may believe that trainee 1 is 1 point higher than average on the continuum because the trainee is “somewhat above average.” Conversely, faculty member B, who also considers the trainee to be “somewhat above average,” believes that “somewhat above average” is commensurate with a 3-point increase on the continuum. Without further grounding, these numbers are seemingly arbitrary.

Instead of comparing individuals to an average, scale points can be grounded by using referents of a standard of quality or performance. This approach is referred to as criterion-based scaling, in which raters are asked to evaluate where an individual falls on a continuum of specific performance referents that all raters are likely to understand (Figure A3).^1,8^ For example, most faculty probably have a good mental model of the level of proficiency they expect from trainees before deeming them ready for unsupervised practice. They also likely can recognize critical deficiencies in a skill set. The spatial measurement differences are preset, with the ability spectrum fixed to a numeric scale: critical deficiencies corresponds to 1, and ready for unsupervised practice corresponds to 7. Cognitively, such an assessment may require a little more thought. The trainee needs to be evaluated against all potentially applicable levels rather than just assessed as better, worse, or about average relative to others. But the resultant scale has fixed numbers that are likely to be similarly interpreted by all faculty.

In norm-referenced scaling, raters are asked to conceptualize a somewhat abstract notion (the average of all trainees, postgraduate year 1s, or some other grouping), while criterion-referenced scaling provides an arguably more systematically recognized referent. In the example in Figure A3, we know that trainee 1 scored an 8 and thus higher than our required score of 7 for being ready for unsupervised practice. We do not immediately know where the trainee stands relative to other trainees; to determine the trainee's rank, we have to do additional calculations.

As these examples demonstrate, a trainee's placement on a continuum is only 1 piece of the puzzle. We must also consider the quality of the data used to make these placements. In the context of applied decisions (eg, who is ready to take care of patients without supervision), we need to also evaluate our information providers (the quality of our faculty ratings). Thus, the scaling methodology must be evaluated in terms of rater reliability (Appendix B).

## Appendix B. Reliability and Intraclass Correlations

Reliability refers to the extent to which measurements are reproducible, consistent, and/or similar. If we ask a patient to step on a scale 3 times, we expect approximately the same value each time.^1^ Conversely, if we observed 3 different values (such as 87 lbs, 205 lbs, and 147 lbs) within a few minutes’ time, this variation would cause us to conclude that our scale, or measurement tool, is not reliable.

If our measurement tool is not working well, we cannot trust the inferences derived from it. Thus, with the 3 example weight measurements, we would not have any certainty about how much the individual actually weighed. Thus, without reliability, there is no validity.^2^ Reliability is an important preliminary indicator to ensure that a measurement tool is working as intended.

### Intraclass Correlation Coefficients

One of the most versatile ways to measure reliability is to use intraclass correlation coefficients (ICCs). They are commonly used to assess reliability in complicated scenarios, such as when the same raters are not rating every individual to be rated.^3^ Depending on the type selected, ICCs allow us to assess the extent to which the same raters are differentially rating individuals, to make inferences about how sample ratings from a pilot study would generalize to a larger sample of ratings, to assess reliability when we have random raters, to assess agreement, and to assess consistency. However, the choice of ICC depends on the type of scenario (or better said, design) you have and how you will use the data.

For our ICC assessment of reliability, we are examining the evaluation ratings received; thus, we are assessing inter-rater reliability. Using the scale analogy from above, we assume that each rater is another measurement. If we have 2 raters rating an individual's performance, we hope that the raters will make similar inferences. If their ratings demonstrate inter-rater reliability, we can assume that our raters are interchangeable and rate independently. In other words, when one rater infers that a trainee is struggling (ie, the trainee truly needs some remediation), we do not need both raters to confirm there is an issue. And vice versa, when one rater infers that a trainee is excellent, we can assume that the trainee is likely truly excellent.

Conversely, differences in scores may be attributable to a wide variety of sources of error, a technical term to explain the unexplained. Applying this concept to an example, assume that 2 faculty members are asked to rate trainee performance on a soft skills training simulation. In this computer-based program, trainees must respond to a series of difficult interpersonal situations. In one scenario, an irate patient feels that his care team has not been communicating well with one another. The trainee is asked to respond to the patient and address several specific contentions the patient presents. Faculty rate the recorded responses for 10 trainees. One of the areas being evaluated is “team communication.” Table B1 shows what the trainees’ scores might look like.

**Table B1.** Example Scores From 2 Faculty Members Showing Complete Consistency, Low Agreement

Trainee	Faculty Member A Score	Faculty Member B Score	Difference Between Raters
Sarah	7	10	3
Kenisha	7	10	3
Tomas	5	8	3
Eduardo	2	5	3
Ricky	6	9	3
Christopher	10	7	3
Emily	7	4	3
Jingwen	6	3	3
Ananya	10	7	3
Zane	8	5	3

Note: On the rating scale, 1 is very poor performance, and 10 is exceptional performance.

In this example, faculty member A and faculty member B do not agree on the scores for any trainees; faculty member B is consistently 3 points higher than faculty member A. Faculty member A may be too lenient or faculty member B may be too strict. However, they both agree on the order (or ranking) of trainees: Christopher and Ananya are the two best and Eduardo the worst on this task. So, both faculty members are consistent with their scores, even if they do not agree on the specific levels. In statistical terms, they have perfect consistency but low agreement.^1^ When we know who specifically is making ratings (eg, faculty member A vs faculty member B) we can use techniques (ie, 2-way random/mixed effects models, ICC [2, ×])^3^ that ignore these specific rater effects. Note that the × refers to the number of estimated raters/evaluations included in the judgment. This approach works well when just knowing who is best and worst on these simulation exercises is sufficient.

Table B2 shows another example of rater scores for 2 faculty members—scores that we would be more likely to observe if we were to actually conduct this study. Our average absolute difference between scores is again 3, as for the example in Table B1, but the scores are now less consistent, so inferences regarding the ranking of our trainees are hard to make. In this scenario, Sarah and Eduardo appear to be the best trainees for faculty member A, whereas Emily and Ananya might be the best for faculty member B.

**Table B2.** Example Scores From 2 Faculty Members With Different Frames of Reference

Trainee	Faculty Member A Score	Faculty Member B Score	Difference Between Raters
Sarah	10	6	–4
Kenisha	4	9	5
Tomas	1	7	6
Eduardo	10	7	–3
Ricky	1	2	1
Christopher	5	5	0
Emily	8	10	2
Jingwen	3	1	–2
Ananya	4	10	6
Zane	9	8	–1

Note: On the rating scale, 1 is very poor performance, and 10 is exceptional performance.

Both faculty members have a wide range of scores; both use the entire continuum from poor to exceptional. But they have some big differences in scores for some of the trainees (eg, a 6-point difference on their perceptions of Ananya's performance). They are in perfect agreement on Christopher, both giving him a 5.

If we were to interview our faculty members, we might get some additional insight. For example, we might find that faculty member A believes that team communication can best be quantified by focusing on how articulate the individual is when communicating with the patient, how much time the trainee spends talking to the patient, the trainee's tone of voice, and the trainee's general clarity of communication. We might find that faculty member B measures team communication by focusing on the trainee's plan of communication rather than the actual communication. For example, this faculty member asks trainees to tell them what they will do to update patients/family members/other team members and then rates the information-sharing plan rather than the actual quality of communication. In this example, the 2 faculty members clearly have different frames of reference for what is being measured, and both likely are tapping into the construct of team communication to some extent. Thus, we might infer that we needed to define team communication for them or provide additional referents so that they better understood what we wanted them to rate.

The design changes for the third example (Table B3). In this scenario, we have random evaluators (eg, any 1 of 20 faculty members made any of the ratings) who rate our candidates. There are 2 administrations: the end of July and the end of August. The trainees are on all different rotations, so many potential faculty members have provided feedback. We cannot correct for their scores. These scores will eventually populate our milestone ratings, so absolute scores matter. In other words, it doesn’t matter if our faculty consistently rated any given trainee highly; it matters where trainees fall on the ability continuum. To be ready for independent practice, a trainee needs to be rated at that level or beyond by the evaluators. Being the best isn’t enough. In this particular case, we need an agreement index. We also need a 1-way random effects model, as we have a random assortment of raters, which is reported as ICC (1, ×). The × refers to the number of estimated raters/evaluations included in the judgment (see below for more details).

**Table B3.** Example Scores From 2 Sets of Random Raters

Trainee	July Faculty Supervisor Score	August Faculty Supervisor Score	Difference Between Administrations
Sarah	7	10	3
Kenisha	9	4	–5
Tomas	5	9	4
Eduardo	1	4	3
Ricky	9	6	–3
Christopher	9	8	–1
Emily	8	5	–3
Jingwen	4	3	–1
Ananya	8	5	–3
Zane	2	6	4

Note: On the rating scale, 1 is very poor performance, and 10 is exceptional performance.

As noted across these examples, when using ICCs, we have several choices to make based on the questions we are answering. In statistical terms, we need to identify whether a 1-way random, a 2-way random, or a 2-way mixed effects design is needed.^3,4^ In most cases when dealing with evaluations, we will need a 1-way random effects model as our judges are random and our milestone scaling requires that the scores (not just rankings) meet a minimal level.^4^

To report ICCs, the first number following ICC represents whether the model is a 1-way or a 2-way model. The second number (represented by the ×) reports how many individuals are estimated to have provided ratings. For the study reported in this paper, we used a 1-way model given that our raters were random. Furthermore, we estimated that a minimum number of 10 evaluations would inform our decisions, so we used ICC (1,10).

## Appendix C. Evaluation Forms for Each Program and Linkage to Competency Area

**Table C1.** Program I: Dartmouth-Hitchcock Internal Medicine Criterion-Referenced Evaluation

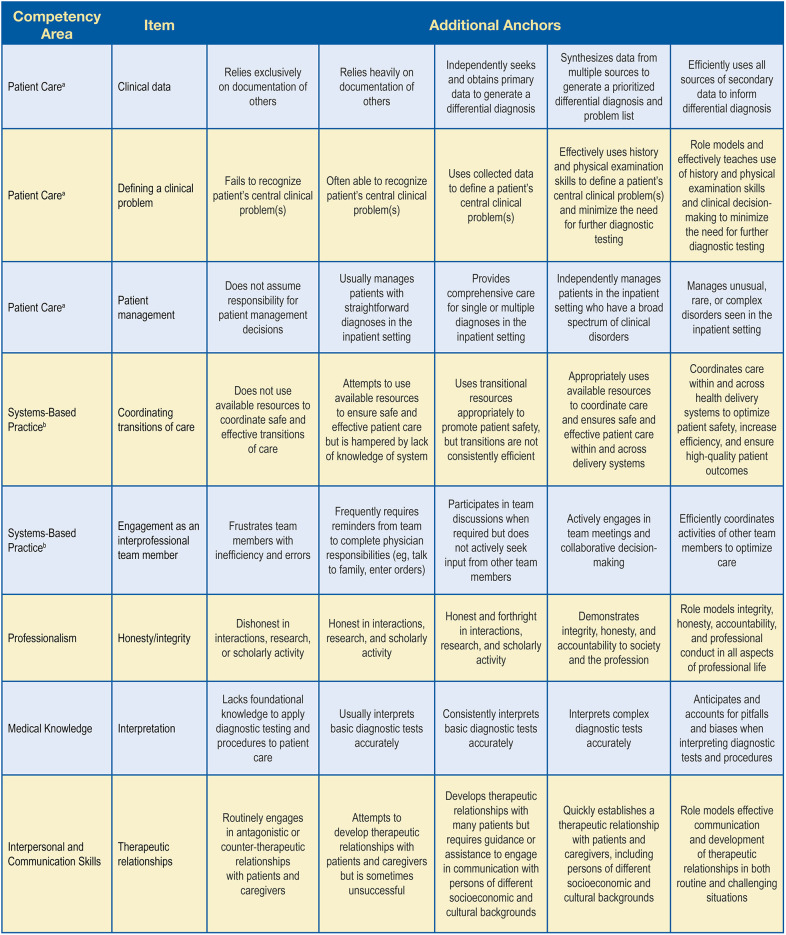

^a^These 3 Patient Care items have been averaged for each evaluation.

^b^These 2 Systems-Based Practice items have been averaged for each evaluation.

Note: Overarching scale anchors: 1 = Critical Deficiencies, 4 = Ready for Unsupervised Practice, 5 = Aspirational (in addition to additional anchors for each item above).

**Table C2.** Program I: Dartmouth-Hitchcock Internal Medicine Norm-Referenced Evaluation

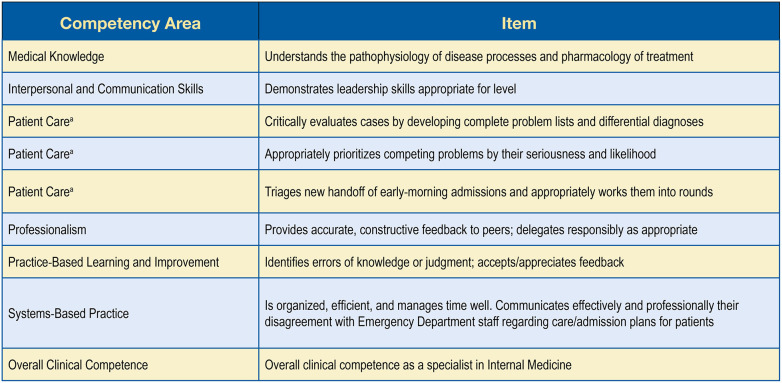

^a^These 3 Patient Care items have been averaged for each evaluation.

Note: Scale anchors: 1-3 = Unsatisfactory, 4-5 = Satisfactory, 6-9 = Superior.

**Table C3.** Program II: University of Texas Southwestern Internal Medicine Criterion- and Norm-Referenced Evaluation

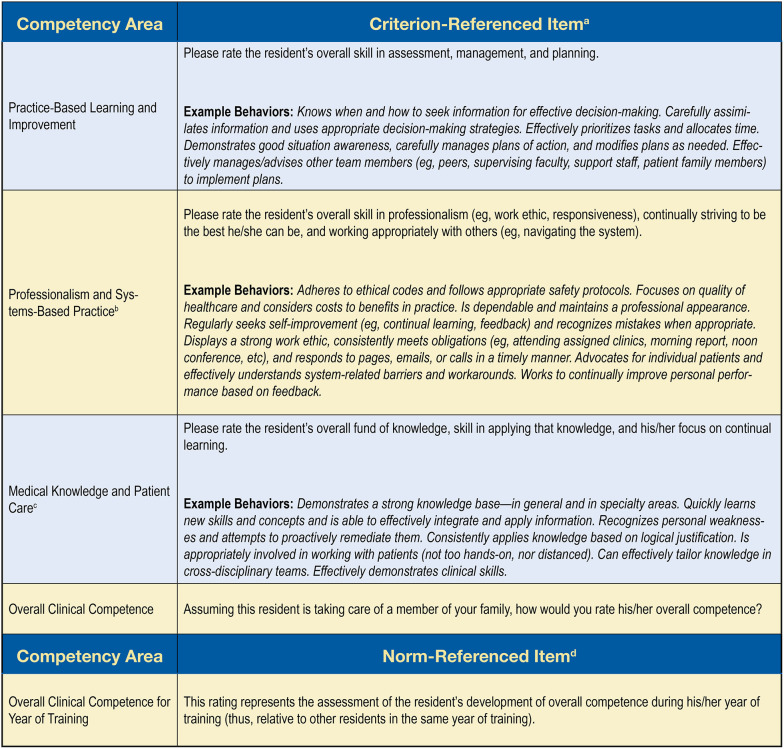

^a^Scale anchors: 1 = Critical Deficiency, 7 = Independent, 9 = Aspirational.

^b^This item was used to represent both content areas.

^c^This item was used to represent both content areas.

^d^Scale anchors: 1-3 = Below Expectations, 4-6 = Meets Expectations, 9 = Exceeds Expectations.

**Table C4.** Program III: University of Texas Southwestern General Surgery Norm-Referenced Evaluation

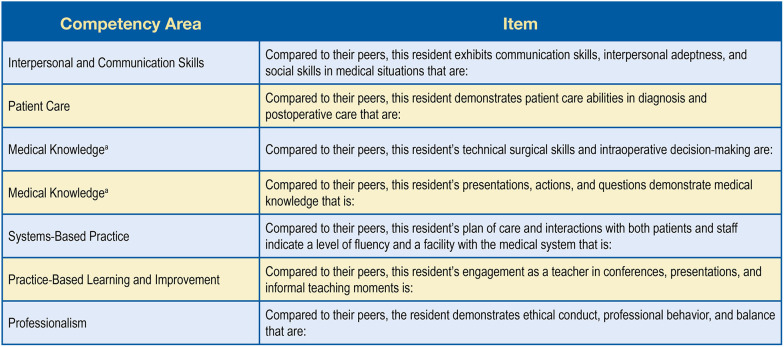

^a^These 2 Medical Knowledge items have been averaged for each evaluation.

Note: Scale anchors: 1 = Satisfactory, 2 = Below Average, 3 = Average, 4 = Above Average, 5 = Excellent.
